# Disparities in Ocular and Periocular Cancer Outcomes: Assessing Survival in Patients of Hispanic Origin

**DOI:** 10.7759/cureus.7713

**Published:** 2020-04-17

**Authors:** Asad Loya, Zainub Abdullah, Aroob Zaheer, Talha Ayaz

**Affiliations:** 1 Medicine, Baylor College of Medicine, Houston, USA; 2 Undergraduate Studies, University of Houston - Main Campus, Houston, USA; 3 Orthopaedic Surgery and Rehabilitation, University of Texas Medical Branch at Galveston, Galveston, USA

**Keywords:** malignancy, cancer, hispanic, ocular, ophthalmic, eye, mortality, periocular, orbital, disparity

## Abstract

A nationwide cancer database was used to perform a retrospective cohort study to compare the overall survival and cause-specific survival in patients with ocular and periocular cancer from varying Hispanic origins. A total of 19,831 cases from the Surveillance, Epidemiology, and End Results (SEER) registries between 1973 and 2015 were obtained for analysis. All-cause and cause-specific mortality risk, with adjustment for age group, sex, race, tumor site, tumor histology, grade, summary stage, laterality, surgery status, radiotherapy status, and chemotherapy status, was examined using Cox proportional hazard models. Of the patients included 19,194 patients were non-Spanish-Hispanic-Latino, and 637 patients were Spanish-Hispanic Latino. The Spanish-Hispanic-Latino population was further subdivided as 398 of Mexican origin, 44 of Puerto Rican origin, 135 of South or Central American (excluding Brazil) origin, and 60 of other Spanish/Hispanic origin (including Europe origin). The mean (+/-SD) follow-up period was 98.57 (+/-93.23) months. In adjusted Cox regression, patients of Spanish-Hispanic-Latino origin demonstrated increased all-cause (HR, 1.173; 95% CI 1.022-1.347; P = 0.023) and cancer-specific mortality (HR, 1.328; 95% CI 1.099-1.604; P = 0.003) as compared to their non-Spanish-Hispanic-Latino counterparts. Upon subclassification by Hispanic origin, patients of Mexican origin had significantly increased all-cause (HR, 1.229; 95% CI 1.032-1.464; P = 0.021) and cancer-specific mortality (HR, 1.516; 95% CI 1.204-1.909; P < .001) and patients with other Hispanic/Spanish origin, including Europe, had significantly increased all-cause (HR, 1.627; 95% CI 1.16-2.28; P =0.005), but not cancer-specific (HR, 1.243; 95% CI 0.734-2.104; P = 0.418) mortality. Patients of Puerto Rican origin and South or Central American (excluding Brazil) origin had no significant difference in all-cause or cancer-specific mortality compared to those of non-Spanish-Hispanic-Latino origin. Mortality risk from ocular and periocular cancers varies extensively by specific Hispanic origin. A greater understanding of these disparities is essential to identify vulnerable populations and provide adequate treatment to optimize long-term outcomes.

## Introduction

The eye and its associated tissues house an amalgam of malignancies ranging from melanoma to lymphoma. Uveal melanoma is the most common intraocular cancer in adults worldwide. It is a subtype of melanoma that involves the ciliary body, iris, or choroid and has a high tendency for metastasis, primarily to the liver [1]. Ocular adnexal lymphoma is a common orbital tumor that encompasses a miscellaneous set of disorders in the lymphoid cells involving the eyelid, conjunctiva, adnexal structures, and orbital soft tissues [2]. The B-cell and T-cell lymphomas originate in the immune system but can also metastasize throughout the body. Orbital and adnexal cancer originate from the skin, muscles, and nerves surrounding the eye. In general, the Hispanic population in the United States have increased impairment of vision as well as worse use of eye care [3]. If left untreated, these malignancies may result in vision loss or death.

Commonly categorized as a single ethnic group and comprising 17% of the population in 2014, Hispanics are the fastest-growing population in the United States [4]. Although varying Hispanic groups share a common language and endure a similar immigration experience, there exists notable heterogeneity in their socioeconomic status, culture, and genetic makeup [5]. Members of shared ancestry and geographical location possess unique risks and may be susceptible to similar diseases. Understanding the diversity of the Hispanic population can lead to greater insight into their genetics.

Numerous studies in the past have shown disparities in outcomes between Hispanic and non-Hispanic groups. However, combining the distinct Hispanic groups as one broad group provides limited explanations on the determinants of such disparities in cancer research. To combat this issue, we conducted a retrospective cohort study to compare overall and cause-specific survival in patients with ocular and periocular cancer from varying Hispanic origins.

## Materials and methods

Cohort

The Surveillance Epidemiology and End Results (SEER) database, a program supported by the National Cancer Institute, was used to obtain case data to perform a retrospective analysis [6]. Since 1973, the SEER registries have regularly obtained statistics on patient demographics, tumor characteristics, treatment administered, and survival of cancer cases reported from 19 different U.S geographic locations. For this study, the International Classification of Diseases for Oncology (ICD-O-3) topography codes for the eye and adnexa (C69.0-9) and eyelid (C44.1) were used to identify all ocular and periocular neoplasms diagnoses between 1973 and 2015. Only primary and malignant neoplasms were included for analysis.

Data from the North American Association of Central Cancer Registries (NAACCR) Hispanic Identification Algorithm (NHIA), an algorithm that combines multiple variables to determine the Hispanic classification, was extracted from SEER to identify the specific Hispanic origin of patients included in the sample. Cases with “NHIA Surname Only” or “Spanish/Hispanic/Latino, NOS” were excluded from the analysis due to limited specificity regarding Hispanic origin. Specific Hispanic origins “Cuban” and “Dominican Republic” were excluded due to case counts less than 20. Cases with missing survival follow-up data and unknown cause-specific death classification were also excluded from the study.

Cases were generally classified first using NHIA codes as “non-Spanish-Hispanic-Latino” or “Spanish-Hispanic-Latino.” Hispanic patients were then further sub-classified through NHIA Hispanic origin code using the following groupings: “Mexican,” “Dominican,” “Puerto Rican,” “South or Central American excluding Brazil,” and “Other specified Spanish/Hispanic Origin including Europe.”

Variables

Extracted demographic variables included age group, race, and sex. Extracted tumor descriptive variables included tumor site, tumor histology, grade, summary stage, and laterality. Treatment variables included the use of surgery, radiotherapy, and chemotherapy. Survival variables included vital status (“alive” or “dead”), cause-specific death classification (“alive or dead of other cause” or “dead attributable to cancer diagnosis”), and survival months.

Analysis

Demographic, tumor, and treatment characteristic variables were tabulated by specific Hispanic origin and Pearson’s chi-squared test was used to compare these variables between groups. All-cause and cause-specific mortality risk were calculated using unadjusted and adjusted multiple Cox regression models for general Hispanic origin and specific Hispanic origin. For all analyses, the non-Hispanic group served as the reference. Covariates for the adjusted model included age group, sex, race, tumor site, tumor histology, grade, summary stage, laterality, surgery status, radiotherapy status, and chemotherapy status. The Kaplan-Meier method was used to calculate five-year and 10-year survival for general and specific Hispanic origin. Statistical significance was determined at p <.05. All statistical computation was conducted using the software IBM Statistical Package for the Social Sciences (SPSS) Statistics version 25 (IBM Corp., Armonk, NY).

## Results

Patient characteristics

Inclusion criteria were met by 19,831 cases, amongst which 19,194 patients were non-Spanish-Hispanic-Latino and 637 patients were Spanish-Hispanic Latino. The Spanish-Hispanic-Latino group was further subdivided as 398 of Mexican origin, 44 of Puerto Rican origin, 135 of South or Central American (excluding Brazil) origin, and 60 of other Spanish/Hispanic origin (including Europe origin). For the overall cohort, the mean (+/-SD) age was 57.02 (+/- 22.91) and the mean follow-up time was 98.57 (+/-93.23) months. The demographics, tumor, and treatment characteristic variables for specific ethnic origins are displayed in Table 1.

**Table 1:** Demographic, tumor, and treatment characteristics of patients with ocular and periocular tumors

		Hispanic Origins										
		Non-Spanish-Hispanic-Latino		Mexican		Puerto Rican		South or Central American excluding Brazil		Other specified Spanish‎/Hispanic origin including Europe		Chi-square
		N	%	N	%	N	%	N	%	N	%	p-value
Age Group	0-59 years	8767	45.7%	248	62.3%	29	65.9%	79	58.5%	28	46.7%	.001
	60-74 years	6146	32.0%	90	22.6%	11	25.0%	37	27.4%	19	31.7%	
	75+ years	4281	22.3%	60	15.1%	4	9.1%	19	14.1%	13	21.7%	
Race	White	16795	87.5%	396	99.5%	44	100%	132	97.8%	60	100%	.001
	Black	934	4.9%	1	0.3%	0	0.0%	3	2.2%	0	0.0%	
	Other	1083	5.6%	1	0.3%	0	0.0%	0	0.0%	0	0.0%	
	Unknown	382	2.0%	0	0.0%	0	0.0%	0	0.0%	0	0.0%	
Sex	Female	9180	47.8%	196	49.2%	27	61.4%	75	55.6%	36	60.0%	0.038
	Male	10014	52.2%	202	50.8%	17	38.6%	60	44.4%	24	40.0%	
Tumor Site	Conjunctiva	2276	11.9%	78	19.6%	3	6.8%	31	23.0%	8	13.3%	.001
	Cornea, NOS	152	0.8%	9	2.3%	2	4.5%	2	1.5%	0	0.0%	
	Retina	1509	7.9%	55	13.8%	7	15.9%	11	8.1%	13	21.7%	
	Choroid	6975	36.3%	81	20.4%	8	18.2%	30	22.2%	16	26.7%	
	Ciliary body	1363	7.1%	12	3.0%	5	11.4%	1	0.7%	1	1.7%	
	Lacrimal gland	765	4.0%	25	6.3%	3	6.8%	15	11.1%	2	3.3%	
	Orbit, NOS	2450	12.8%	70	17.6%	6	13.6%	30	22.2%	6	10.0%	
	Overlapping lesion of eye and adnexa	245	1.3%	2	0.5%	0	0.0%	1	0.7%	1	1.7%	
	Eye, NOS	896	4.7%	12	3.0%	3	6.8%	2	1.5%	4	6.7%	
	Eyelid	2563	13.4%	54	13.6%	7	15.9%	12	8.9%	9	15.0%	
Cancer Histology	Adenomas and adenocarcinomas	360	1.9%	8	2.0%	1	2.3%	4	3.0%	3	5.0%	.001
	Adnexal and skin appendage neoplasms	965	5.0%	18	4.5%	3	6.8%	4	3.0%	3	5.0%	
	Cystic mucinous and serous neoplasms	176	0.9%	1	0.3%	0	0.0%	0	0.0%	0	0.0%	
	Nevi and melanomas	10597	55.2%	138	34.7%	16	36.4%	42	31.1%	27	45.0%	
	Malignant lymphomas	3719	19.4%	94	23.6%	10	22.7%	44	32.6%	9	15.0%	
	Other	3377	17.6%	139	34.9%	14	31.8%	41	30.4%	18	30.0%	
Grade	Grade I‎/II	1286	6.7%	39	9.8%	5	11.4%	14	10.4%	4	6.7%	.001
	Grade III‎/IV	827	4.3%	39	9.8%	3	6.8%	6	4.4%	4	6.7%	
	Lymphomas	2815	14.7%	79	19.8%	9	20.5%	36	26.7%	7	11.7%	
	Unknown	14266	74.3%	241	60.6%	27	61.4%	79	58.5%	45	75.0%	
Summary stage	Localized	2659	13.9%	66	16.6%	8	18.2%	35	25.9%	8	13.3%	.001
	Regional	194	1.0%	10	2.5%	0	0.0%	1	0.7%	0	0.0%	
	Distant	424	2.2%	12	3.0%	0	0.0%	5	3.7%	1	1.7%	
	Unknown‎/unstaged	15917	82.9%	310	77.9%	36	81.8%	94	69.6%	51	85.0%	
	In situ	0	0.0%	0	0.0%	0	0.0%	0	0.0%	0	0.0%	
Laterality	Unilateral	18235	95.0%	373	93.7%	41	93.2%	129	95.6%	54	90.0%	0.268
	Bilateral	680	3.5%	23	5.8%	3	6.8%	5	3.7%	5	8.3%	
	Paired site, but unknown laterality	246	1.3%	2	0.5%	0	0.0%	1	0.7%	1	1.7%	
	Not a paired site	33	0.2%	0	0.0%	0	0.0%	0	0.0%	0	0.0%	
Radiation Status	No‎/Unknown	11234	58.5%	260	65.3%	34	77.3%	75	55.6%	42	70.0%	0.002
	Yes	7960	41.5%	138	34.7%	10	22.7%	60	44.4%	18	30.0%	
Surgery Status	Not performed	7977	41.6%	129	32.4%	7	15.9%	53	39.3%	16	26.7%	.001
	Performed	10917	56.9%	264	66.3%	35	79.5%	79	58.5%	43	71.7%	
	Unknown	300	1.6%	5	1.3%	2	4.5%	3	2.2%	1	1.7%	
Chemotherapy status	No‎/Unknown	17100	89.1%	321	80.7%	36	81.8%	117	86.7%	47	78.3%	.001
	Yes	2094	10.9%	77	19.3%	8	18.2%	18	13.3%	13	21.7%	

Survival outcomes

After adjustment for confounding covariates, Spanish-Hispanic-Latino patients had significantly increased all-cause (HR, 1.173; 95% CI 1.022-1.347; P = 0.023) and cancer-specific mortality (HR, 1.328; 95% CI 1.099-1.604; P = 0.003) compared to their non-Spanish-Hispanic-Latino counterparts. In an adjusted sub-analysis compared to a non-Spanish-Hispanic-Latino background, patients of Mexican origin had significantly increased all-cause (HR, 1.229; 95% CI 1.032-1.464; P = 0.021) and cancer-specific mortality (HR, 1.516; 95% CI 1.204-1.909; P < .001) and patients with other Hispanic/Spanish origin, including Europe, had significantly increased all-cause (HR, 1.627; 95% CI 1.16-2.28; P =0.005) but not cancer-specific (HR, 1.243; 95% CI 0.734-2.104; P = 0.418) mortality. Patients with Puerto Rican origin and South or Central American (excluding Brazil) origin had no significant difference in all-cause or cancer-specific mortality compared to those of non-Spanish-Hispanic-Latino origin (Table 2). Kaplan-Meier survival rates at 5- and 10-year follow-up are displayed in Table 3.

**Table 2:** Unadjusted and adjusted Cox regression analysis

		Overall survival		Cause-specific survival	
		HR (95% CI)	p-value	HR (95% CI)	p-value
Unadjusted analysis	Non-Spanish-Hispanic-Latino	reference	reference	reference	reference
	Hispanic	0.916 (0.798, 1.05)	0.207	0.997 (0.827, 1.203)	0.978
Adjusted analysis	Non-Spanish-Hispanic-Latino	reference	reference	reference	reference
	Hispanic	1.173 (1.022, 1.347)	0.023*	1.328 (1.099, 1.604)	0.003*
Unadjusted analysis	Non-Spanish-Hispanic-Latino	reference	reference	reference	reference
	Mexican	0.952 (0.8, 1.133)	0.581	1.113 (0.885, 1.398)	0.36
	Puerto Rican	1.036 (0.652, 1.645)	0.881	1.293 (0.716, 2.337)	0.395
	South or Central American excluding Brazil	0.565 (0.393, 0.814)	0.002*	0.515 (0.299, 0.888)	0.017*
	Other specified Spanish/Hispanic Origin including Europe	1.348 (0.963, 1.888)	0.082	1.152 (0.682, 1.947)	0.596
Adjusted analysis	Non-Spanish-Hispanic-Latino	reference	reference	reference	reference
	Mexican	1.229 (1.032, 1.464)	0.021*	1.516 (1.204, 1.909)	.001
	Puerto Rican	1.454 (0.915, 2.312)	0.113	1.671 (0.923, 3.025)	0.09
	South or Central American excluding Brazil	0.711 (0.494, 1.025)	0.067	0.734 (0.425, 1.266)	0.266
	Other specified Spanish/Hispanic Origin including Europe	1.627 (1.16, 2.28)	0.005*	1.243 (0.734, 2.104)	0.418

**Table 3:** Kaplan-Meier 5-year and 10-year survival rates.

		Overall Survival		Cause-specific survival	
		5yr survival	10yr survival	5yr survival	10yr survival
General	Non-Spanish-Hispanic-Latino	73.7%	57.5%	83.6%	75.5%
	Hispanic	76.4%	61.4%	85.1%	78.0%
Specific Origin	Non-Spanish-Hispanic-Latino	73.7%	57.5%	83.6%	75.5%
	Mexican	75.2%	62.9%	84.6%	76.1%
	Puerto Rican	76.3%	58.5%	81.7%	72.6%
	South or Central American Excluding Brazil	85.8%	70.5%	91.1%	87.7%
	Other specified Spanish/Hispanic Origin Including Europe	66.1%	42.1%	79.2%	73.5%

## Discussion

There is appreciable heterogeneity in ocular and periocular mortality within the Hispanic population. The Spanish-Hispanic-Latino population had greater all-cause and cancer-specific mortality than their non-Spanish-Hispanic-Latino counterparts. Through further analysis, we found significant differences in survival by specific Spanish-Hispanic-Latino origin.

Relative to non-Spanish-Hispanic-Latino patients, patients of Mexican origin demonstrated increases in both cancer-specific and all-cause mortality. Patients of Puerto Rican origin and South or Central American (excluding Brazil) origin demonstrated no difference in all-cause and cancer-specific mortality relative to non-Spanish-Hispanic-Latino. Importantly, although patients of other Hispanic/Spanish origin, including Europe, demonstrated an increase in all-cause mortality, they possessed no difference in cancer-specific mortality; this finding may be explained due to an increase in mortality unrelated to the primary cancer itself (i.e., cardiovascular disease). Our results demonstrate that Hispanic subgroups possess differing levels of disease burdens, likely due to multiple factors, including genetic heterogeneity and health disparities. 

Our findings are consistent with other studies of varying anatomical regions and cancer histology, which found that the Hispanic population is at higher risk for malignancies and related complications (i.e., mortality) [7-10]. Studies such as the Los Angeles Latino Eye Study have attempted to quantify risk factors for ocular conditions in the Hispanic population at large, but sparse literature exists on the effect of genetic and ancestral variation on such conditions, specifically ocular and periocular malignancies [11]. Our study highlights which of the specific subsets of the Hispanic population are most vulnerable to disparities in ocular and periocular neoplasms. We did not find significant differences in all-cause and cause-specific mortality in the Puerto Rican origin populations as found by previous studies [12]. Risk factors for lung, colon, and hepatic malignancies addressed by these studies such as smoking and alcohol use are more prevalent in the Puerto Rican origin population in relation to other Hispanic subgroups [12-13]. However, risk factors for uveal melanoma, including fair skin, are not significantly increased in this subset [14]. Ocular adnexal lymphoma risk factors, such as certain infectious agents, have not been studied in the American Hispanic population [15]. Given the genetic heterogeneity between Hispanic populations of varying origin, when these patients develop ocular and periocular malignancies, they may be genetically predisposed to less advanced disease, and, therefore, possess lower mortality related to the disease [1-2]. These key differences between ophthalmologic malignancies and non-ophthalmologic malignancies may account for differences in our findings regarding patients of Puerto Rican Hispanic origin.

It is possible that our findings may have been influenced by the lower socioeconomic status of the Spanish-Hispanic-Latino population of the U.S. in comparison to the non-Spanish-Hispanic-Latino population, a variable which the database is limited in accounting for [16]. However, additional insight can be offered by prior studies. Higher levels of education and socioeconomic status are indicators of general eye health knowledge, crucial for the early detection of malignancies [3,17]. Among the groups studied, the Puerto Rican origin subset has the highest rates of completion of high school [16]. In contrast, individuals of Mexican origin in the U.S. have been found to have 5% and 31.2% lower high school completion rates than all Hispanic groups and non-Hispanic white groups respectively [16]. Level of education may, therefore, contribute to the lack of significant difference in all-cause and cause-specific mortality in patients of Puerto Rican origin as well as the increased all-cause and cause-specific findings in patients of Mexican origin.

Our findings may also reflect disparities in healthcare access (i.e. adherence to routine preventative eye care). The Hispanic population is more than twice as likely to be uninsured than the non-Hispanic white population, placing a great strain on access to needed early interventions [18]. Compared to non-Hispanic whites, Hispanics had lower levels of glycated hemoglobin (HbA1c) testing, eye exams, and other preventative health screenings [19]. Compared to patients of Central/South American origin, patients of Mexican origin had lower levels of preventative cancer screening for cervical (3% lower), breast (6% lower), and colorectal (6% lower) cancers [13]. Similarly, decreases were found when comparing screening test use in patients of Mexican origin to those of Puerto Rican origin for breast (2% lower), cervical (6% lower), and colorectal (16% lower) cancers [13]. Patients who receive decreased age-specific preventative cancer screening are likely to have decreased healthcare access and, therefore, may have disease detected at more advanced stages and poorer adherence to treatment regimens.

The Hispanic population in the United States consists of diverse subcategories that may be subject to various genetic predispositions tracing back to common ancestry and origins of location [5,20-21]. This may manifest beyond the scope of malignancies as well [22]. It is important to identify these differences with regard to both ophthalmological and broader health conditions to effectively treat this rapidly growing demographic. Focusing future studies on specified population parameters will help us understand the factors behind the failure or success of treatment. As the treatment of malignancies is shifting toward targeted genetic therapies, it is becoming increasingly crucial to further analyze the demographic nature of these abnormalities.

Our study encountered multiple limitations. As the study utilized a national database, we are reliant on the data that is contained within it. Several variables, such as stage and grade, were not available for all patients included in the study, which may have confounded our findings. SEER also does not provide individual-level data regarding socioeconomic status (SES), education attainment, comorbidities, and modifiable risk factors (i.e., obesity, smoking), limiting our ability to account for those variables; identifying high-risk populations is still an important first step, however. Importantly, despite using a national database, our study was limited in sample size, which led to the exclusion of important subgroup origins (i.e. Cuban origin). Our study may also be limited by potential type II errors, causing real differences to not be detected. Due to the nature of our study, additional limitations included disease progression from the presentation, limited treatment data, and a possibility for miscoding. Despite these limitations, however, the SEER database has been validated for such studies.

## Conclusions

Mortality risk following ocular and periocular cancer diagnoses varies extensively by Spanish-Hispanic-Latino sub-ethnicity when compared to non-Spanish-Hispanic-Latino patients. Understanding differences in disease manifestation is important to identify vulnerable populations and develop effective management plans in order to optimize long-term patient outcomes.
